# A Multimodal Intervention Including Tirzepatide in Women with Lipedema and Excess Body Weight: A Pilot Case Series

**DOI:** 10.3390/jcm15218643

**Published:** 2026-11-06

**Authors:** Karolina Kędzierska-Kapuza, Judyta Samul-Jastrzębska, Monika Cygler, Magdalena Jerzak, Edward Franek

**Affiliations:** 1 Department of Internal Medicine, Endocrinology and Diabetology, National Medical Institute of the Ministry of Interior Affairs and Administration in Warsaw, 137 Wołoska Street, 02-507 Warsaw, Poland; 2 Doppler Institute—Warsaw Vascular Institute, 24/U4 Joseph Conrad Street, 01-922 Warsaw, Poland; 3 Magdalena Jerzak Sole Proprietorship, Zełwągi 32, 11-730 Mikołajki, Poland

**Keywords:** lipedema, tirzepatide, low-carbohydrate diet, obesity, subcutaneous adipose tissue, quality of life

## Abstract

**Background:** Lipedema is a chronic adipose tissue disorder characterized by disproportionate lower-limb fat accumulation, pain, and resistance to conventional weight-loss strategies. Tirzepatide produces substantial weight loss, but evidence in lipedema is limited. **Methods:** This single-center ambispective pilot case series described a 22–25-week routine clinical course in 2025. During the first 8–9 weeks, all participants received only a low-carbohydrate Mediterranean diet and recommendations for aerobic and resistance exercise; no participant received tirzepatide during this phase. After completion of the 8–9-week non-pharmacological phase, four participants started tirzepatide (2.5–7.5 mg once weekly) and continued it for the remaining 14–16 weeks, whereas one participant declined pharmacotherapy. After approval by the Bioethics Committee (Resolution No. 12/2026, 4 February 2026), the 2025 data were analyzed retrospectively; participants gave verbal consent to further follow-up in March–April 2026 and were followed up prospectively, and all signed written consent for publication of their data and photographs in October 2026. Anthropometric, ultrasound, biochemical, pain, and quality-of-life outcomes were summarized descriptively for each case. Where available, changes are reported separately for the diet-and-exercise phase (T1–T2) and the tirzepatide phase (T2–T3). **Results:** Weight loss among the five completers ranged from 7.7 to 17.1 kg. Body fat percentage decreased in four of five participants, whereas skeletal muscle mass decreased numerically in all four tirzepatide-treated participants. Lower-limb circumferences and subcutaneous adipose tissue thickness generally decreased, although the magnitude and distribution of changes differed between participants. Fasting glucose and HOMA-IR decreased in most cases, whereas lipid changes were mixed. Patient-reported SF-36 scores (assessed only at T2 and T3) generally improved among the four participants receiving tirzepatide, particularly for bodily pain, while pressure pain threshold responses (T2 to T3) were heterogeneous. No adverse events, gastrointestinal symptoms, dose reductions, treatment interruptions, discontinuations, or serious adverse events were reported by the four tirzepatide-treated participants; the study was not designed to assess safety. **Conclusions:** This ambispective pilot case series provides preliminary, hypothesis-generating observations on a multimodal intervention that included tirzepatide in four of five women with lipedema and excess body weight. The observed changes cannot be attributed to tirzepatide or interpreted as a lipedema-specific effect because all participants received concurrent dietary, exercise, and supplementation interventions, and the participant who declined pharmacotherapy was not a comparator or control.

## 1. Introduction

Lipedema is a chronic, painful disorder of subcutaneous adipose tissue that predominantly affects women. It is characterized by symmetrical, disproportionate fat accumulation in the lower limbs, tenderness, easy bruising, and relative resistance of affected tissue to weight loss [1,2]. Overweight or obesity commonly coexists and may worsen mobility, symptoms, and cardiometabolic risk [3,4].

Current management is primarily symptomatic and includes compression, complex decongestive therapy, exercise, dietary intervention, and, in selected patients, lymph-sparing liposuction [2,5,6,7,8,9,10,11,12,13]. Conservative therapies may reduce edema, pain, and functional limitations but do not consistently reduce pathological adipose tissue, whereas liposuction is invasive, costly, and not widely available. No pharmacological treatment is currently approved specifically for lipedema [2,5,6].

Metabolic abnormalities, including insulin resistance, have been described in women with lipedema, particularly when obesity is present [3,14]. Low-carbohydrate and ketogenic diets may support weight reduction and symptom improvement [11,12], while incretin-based therapies offer a potential pharmacological approach. Preliminary reports with exenatide and mechanistic discussions of tirzepatide suggest possible benefits, but clinical evidence remains sparse [15,16].

We therefore evaluated changes in anthropometric, metabolic, ultrasound, pain, and quality-of-life outcomes during a 22–25-week clinical course comprising an initial 8–9-week phase of low-carbohydrate Mediterranean diet and physical activity alone, followed by a 14–16-week phase in which four participants additionally received tirzepatide. The study was designed as an exploratory pilot case series to generate hypotheses for future controlled trials.

## 2. Materials and Methods

### 2.1. Study Design and Participants

This single-center ambispective pilot case series describes a 22–25-week clinical course comprising an initial 8–9-week period of routine clinical care with dietary intervention and physical activity alone, followed by a 14–16-week period in which four participants additionally received tirzepatide. The entire course took place in 2025 as part of routine clinical care at the Individual Specialist Medical Practice Karolina Kędzierska-Kapuza (Warsaw, Poland). The data from this course (T1–T3, the main outcomes) were analyzed retrospectively, and the participants were followed up prospectively thereafter; ethics approval and consent are described in Section 2.2. Eight adult women were screened through an online questionnaire distributed in lipedema-focused social media groups. A phlebologist assessed eligibility using the Allen–Hines clinical criteria and the S2k guideline [2]. Lipedema was confirmed in seven women; one screened woman was not included because the diagnosis remained uncertain. Two eligible women discontinued follow-up because they were unable to travel to the clinic for visits scheduled at predefined time points. Five women completed follow-up and were included in the descriptive analysis. The data of the two women who discontinued follow-up were not used. No participant who completed follow-up was excluded from the analysis. The participant flow is shown in Figure 1.

Clinical eligibility criteria were female sex, age ≥ 18 years, clinically diagnosed lipedema, and concomitant overweight or obesity. All participants had a BMI > 27 kg/m^2^ with at least one obesity-related complication and therefore had an established clinical indication for pharmacological obesity treatment. Exclusion criteria assessed before the initial non-pharmacological phase included previous treatment with a GLP-1 or dual GIP/GLP-1 receptor agonist, pregnancy or breastfeeding, lymphedema, active cancer, multiple endocrine neoplasia type 2 (MEN2), personal or family history of medullary thyroid carcinoma, history of pancreatitis, chronic kidney disease, heart failure, liver failure, or another condition likely to interfere with metabolic or body-composition assessment. These criteria were applied during routine clinical assessment before the start of the dietary intervention.

The clinical data analyzed in this report were collected between 5 April 2025 and 27 September 2025. The outcomes analyzed were those assessed routinely at T1, T2, and T3 (body weight and body composition, central and lower-limb circumferences, ultrasound SAT thickness, fasting metabolic and laboratory parameters, pressure pain threshold, and SF-36 domain scores); they were specified in the protocol submitted to the Bioethics Committee before data analysis, and no single primary endpoint was defined. No formal sample-size calculation was performed; the series comprises all eligible women who completed the clinical course, consistent with the exploratory, hypothesis-generating purpose of a pilot case series. The protocol is available from the corresponding author on reasonable request.

### 2.2. Study Timeline, Ethics, and Regulatory Context

All participants attended the clinic as part of routine medical care in 2025. For the first 8–9 weeks, all participants received exclusively an individually prescribed low-carbohydrate Mediterranean diet and recommendations for regular aerobic and resistance exercise. Tirzepatide was not administered during this initial phase. Therapeutic recommendations were based solely on individual clinical indications and standard clinical practice.

All procedures described in this report—the online intake questionnaire, phlebological assessment, anthropometric and bioelectrical impedance measurements, ultrasound, laboratory tests, pressure algometry, the SF-36 questionnaire, clinical photographs, dietary and physical-activity prescription, and the decision to start tirzepatide within its approved obesity indication—were performed as part of routine clinical care.

During the clinical course, at T2 (week 8–9), four participants started tirzepatide within its approved obesity indication; one participant declined pharmacotherapy and continued diet and physical activity alone. Follow-up continued to T3 (week 22–25), and the T3 assessments took place on 13 September 2025 (Participants 1, 3, and 4) and 27 September 2025 (Participants 2 and 5).

Ethics approval and consent. The clinical course (5 April–27 September 2025) was delivered as routine clinical care, and treatment decisions were based on clinical indications. Formal ethics approval was obtained after completion of the clinical course: the study protocol, which described the completed course and requested approval for the analysis and publication of these data and for further follow-up of the participants, was approved by the Bioethics Committee of the National Medical Institute of the Ministry of the Interior and Administration on 4 February 2026 (Resolution No. 12/2026). After approval, all participants gave verbal consent to further follow-up at follow-up visits between 17 March and 29 April 2026, as recorded in the medical documentation (Table 1). In October 2026, before publication, all participants signed written informed consent for the use and publication of their clinical data and photographs.

Participant-level dates of the clinical assessments, tirzepatide initiation, and verbal consent to follow-up are given in Table 1.

### 2.3. Anthropometric Measurements and Body Composition

Assessments were scheduled at T1 (week 0, start of the diet-and-exercise-only phase), T2 (week 8–9, after the non-pharmacological phase and immediately before tirzepatide initiation), and T3 (week 22–25, end of follow-up), as applicable to each outcome. Body weight and body composition were assessed using an InBody 570 bioelectrical impedance analyzer (InBody Co., Seoul, Republic of Korea), including skeletal muscle mass, fat mass, fat-free mass, BMI, body fat percentage, extracellular water ratio, and segmental composition. All bioelectrical impedance measurements were performed in participants who were fasting and had voided immediately beforehand, to standardize hydration status; measurement times ranged from 09:09 to 13:21. Waist-to-hip ratio and waist-to-height ratio were calculated [17]. A trained physiotherapist measured the circumference of the lower limbs using a non-elastic tape measure at standardized anatomical points using the segmental technique. The same physiotherapist performed all circumference measurements at T1, T2, and T3. This technique identifies eight anatomical landmarks, which are identical to those used in measurements for custom-made compression garments.

The first point identified was the narrowest one above the ankle (point cB). Given the impossibility of objectively identifying the gastrocnemius muscle insertion without ultrasound, the calf muscle insertion point (point B1) was identified as an intermediate point between points B and C. The third point (cC) is the thickest point of the calf muscle, identified with the patient in an orthostatic position with the limbs extended. The fourth point (point cD) is at the level of the styloid process of the head of the fibula. The fifth point (cE) was measured in the popliteal fossa with the limb flexed at approximately 30°. The sixth point (cF) is two-thirds of the thigh height. The seventh point (cG) is below the gluteal crease. In the original description of the segmental technique [18], point E1 serves an auxiliary function—it is determined solely to divide the thigh height (segment E–G) into three equal parts and thus locate point F (two-thirds of the height). The authors themselves do not describe it as an independent clinically reported measurement site, but rather as an intermediate step in the geometric construction. Therefore, in the clinical protocol, we decided to omit E1 as the point with the lowest clinical value of the eight described, while retaining the remaining seven (cB, cB1, cC, cD, cE, cF, cG), which shortened the examination time and reduced the patient’s workload (Figure 2). Measurements were performed twice on each limb in the orthostatic position. The two measurements on each limb were averaged, and the value reported for a given point in the analysis was the mean of the right- and left-limb averages (e.g., cB value = (cB right + cB left)/2). This approach reflects the typically symmetrical nature of adipose tissue involvement in lipedema and allows for a single, representative value per participant at each time point. Potential interlimb differences (asymmetries) were not analyzed separately, which is a limitation of this approach [18]. Waist circumference was measured with a non-elastic tape, without pressure, midway between the lower rib margin (the last palpable rib) and the upper iliac crest, at the end of normal exhalation. The patient was standing with her legs apart. Hip circumference was measured at the widest point around the buttocks, at the level of the greater trochanters of the femurs, standing, with the feet together.

Clinical photographs were obtained at T1 and T3 in a standing position (anterior and posterolateral views), with participants wearing underwear, against the same background; camera distance and lighting were not formally standardized. Photographs are presented for illustration only and were not used for quantitative assessment.

### 2.4. Ultrasound Measurement

All participants underwent lower-limb venous duplex ultrasonography and measurement of subcutaneous adipose tissue (SAT) thickness using a 4–12 MHz linear transducer (Philips ClearVue 350). The probe was positioned perpendicular to the skin without compression, as described by Amato et al. [19]. SAT was measured bilaterally at the anterior thigh, pretibial region, lateral calf, and 6 cm above the medial malleolus [20]. Each measurement was repeated three times and averaged. SAT thickness was reported in millimeters, separately for the right and left limb (upper and lower values for each site). All examinations were performed by the same sonographer (J.S.-J.), who was not blinded to the time point; intra- and inter-rater reliability were not assessed. Ultrasound assessments used for the main pre-post comparison were performed at T1 (week 0) and T3 (week 22–25). Ultrasound was not repeated at T2; therefore, SAT changes cannot be apportioned between the diet-and-exercise and tirzepatide phases.

### 2.5. Biochemical Analyses

After a 12 h overnight fast, blood and morning urine samples were obtained at T1 (week 0) and T3 (week 22–25). Measurements included complete blood count, lipid profile, high-sensitivity C-reactive protein, alanine aminotransferase, creatinine, thyroid-stimulating hormone, 25-hydroxyvitamin D, fasting glucose, fasting insulin and urine albumin-to-creatinine ratio. HOMA-IR was calculated from fasting glucose and insulin.

### 2.6. Dietary Intervention

During the first 8–9 weeks, diet and physical activity were the only therapeutic interventions. Participants followed an individually prescribed low-carbohydrate Mediterranean diet supervised by a clinical dietitian. Energy intake was based on body composition and activity level, with a planned deficit of 300–500 kcal/day. Carbohydrates provided 18–25% of energy, protein 16–21%, and fat 54–66%, with a target of 25 g/day of dietary fiber. At the prescribed energy intakes, the protein target corresponded to approximately 90–100 g/day (approximately 0.8–1.4 g/kg actual body weight at T2); protein intake was not specifically increased after tirzepatide initiation. The diet emphasized fish, poultry, eggs, legumes, lean dairy, unsaturated fats, whole-grain products, vegetables, and fruit. Vitamin D3 at 4000 IU/day and omega-3-6-9 supplementation (Love Life Kobieta oil, 3 g/day, including 2 g/day of EPA+DHA) were continued throughout follow-up. Vitamin D3 and the omega-3-6-9 oil were purchased directly by the participants; the study received no external funding for medications or supplements. The dietary regimen was formulated to emphasize sources of monounsaturated and omega-3 fatty acids while limiting highly processed foods and maintaining an appropriate balance of polyunsaturated fatty acids. The planned diet, including supplementation, provided approximately 6.8 g/day of polyunsaturated fatty acids in total; the omega-3-6-9 supplement (3 g/day of oil, including 2 g/day of EPA+DHA) contributed approximately 2–3 g/day of this amount. Adherence was monitored by the study dietitian during regular video consultations and by review of dietary records. As an illustrative example, a representative day tracked by one participant using a diet-monitoring application totaled 1854 kcal (target 1850 kcal), with 120.4 g carbohydrate (target 116 g), 118.2 g fat (target 113 g), 89.4 g protein (target 93 g), and 28.3 g fiber (target 25 g), illustrating adherence to the prescribed macronutrient targets.

### 2.7. Functional and Physiotherapeutic Assessments

Participants were advised to perform regular aerobic activity and resistance exercise at least twice weekly from T1 onward. Adherence to physical-activity recommendations was reviewed during regular video consultations and assessed using participant-completed activity diaries. The number of resistance-training sessions recorded in the diaries during the tirzepatide phase was two per week. All participants declared that they performed exercises while wearing compression garments they already owned—toeless compression tights (circular or flat knit, depending on the stage and clinical picture of disease). Pressure pain threshold was measured using a handheld Force Dial FDK/FDN Series algometer (Wagner Instruments) with a 1 cm^2^ transducer; higher values indicated lower pressure sensitivity [21]. This was consistent with the design validated by Kinser et al. [22], who demonstrated good reliability and validity of this type of device relative to a force platform and emphasized that a constant rate of increase in manual force is important for measurement reliability. Force was applied perpendicularly to the skin with a constant rate of increase. Participants were instructed to report the moment at which the pressure sensation transformed into pain. The order of measurement was constant across the seven measurement points (from cB to cG). Two consecutive measurements were taken 30 s apart, and the results were averaged. All measurements at a given time point were performed by the same experienced observer. Pressure pain threshold was assessed only at T2 and T3.

Health-related quality of life was assessed using the Polish version of the SF-36v2 questionnaire [23] developed and psychometrically validated by the Central Institute for Labor Protection—National Research Institute (CIOP-PIB), based on the official translation from the IQOLA project. Responses were scored according to the algorithm described in the CIOP-PIB manual for the SF-36v2 questionnaire. Raw responses were recoded according to the scoring key and then items within the subscales were averaged. This resulted in a score ranging from 0 to 100 for each of the eight domains, with higher scores indicating better health. Participants completed the questionnaire independently at T2 (week 8–9, immediately before tirzepatide initiation) and T3 (week 22–25). The effect of the 8–9-week dietary and exercise phase alone on quality of life was not assessed separately because the SF-36 was not administered at T1 [24,25]. Consequently, the SF-36 results describe only the 14–16-week period between T2 and T3; they cannot be used to evaluate the diet-and-exercise phase or the full multimodal course, and changes from T2 to T3 cannot be attributed to tirzepatide alone.

### 2.8. Pharmacological Intervention

At T2 (week 8–9), after completion of the diet-and-exercise-only phase, four participants started tirzepatide according to the standard dose-escalation schedule in the Summary of Product Characteristics: 2.5 mg once weekly for 4 weeks, followed by 5 mg once weekly for 4 weeks and 7.5 mg once weekly for the remaining 6–8 weeks, until T3 (week 22–25). Tirzepatide exposure therefore lasted 14–16 weeks. The starting, maximum, and final doses were 2.5, 7.5, and 7.5 mg, respectively, in each treated participant. One participant declined pharmacotherapy and continued diet and physical activity alone. Because treatment allocation was not randomized and only one participant did not receive tirzepatide, this participant was treated as an individual case rather than as a control. Tirzepatide was obtained on private prescription and purchased by the participants themselves, consistent with routine clinical care for an approved obesity indication; it was not provided or funded by the study.

### 2.9. Safety Monitoring

From tirzepatide initiation at T2 (week 8–9) until T3 (week 22–25), participants were asked at each follow-up contact (during the regular video consultations and clinic visits) about adverse events, including gastrointestinal symptoms, and treatment interruptions or dose modifications were recorded. At every contact, participants were specifically asked about nausea, vomiting, diarrhea, constipation, abdominal pain, dyspepsia, decreased appetite, injection-site reactions, and symptoms suggestive of hypoglycemia or gallbladder disease. No adverse events were reported among the four tirzepatide-treated participants; specifically, no nausea, constipation, or other gastrointestinal symptoms were reported. No dose reductions, temporary interruptions, permanent discontinuations, or serious adverse events were recorded. Given the small sample size and short exposure, these observations do not establish the safety of tirzepatide in this population.

### 2.10. Statistical Analysis

Given the small sample size and the descriptive case-series design, no inferential statistical testing was performed. Continuous variables are reported as individual values and, where helpful for summary purposes, as means, standard deviations, ranges, or percentage changes. These summaries were not used for between-group comparisons or hypothesis testing. All findings should be considered exploratory and hypothesis-generating. No p-values or confidence intervals are reported. Terms such as “decrease”, “increase”, or “improvement” refer to numerical changes in individual values and do not imply statistical significance. Changes are presented as absolute (and, where stated, percentage) differences for T1–T2 (diet-and-exercise phase), T2–T3 (tirzepatide phase in Participants 1–4), and T1–T3.

## 3. Results

Five women completed the 22–25-week clinical course. During weeks 0 to 8–9, all five received diet and physical activity alone. At T2 (week 8–9), Participants 1–4 started tirzepatide and continued it for 14–16 weeks in addition to the non-pharmacological intervention, whereas Participant 5 declined pharmacotherapy and continued diet and physical activity alone. The participants differed in age, lipedema stage, venous disease (Table 2), and baseline metabolic profile; therefore, Participant 5 was not considered a control or comparator. Weight loss from T1 to T3 ranged from 7.7 to 17.1 kg. Between T1 and T2, body weight changed by +1.5, −5.3, +1.6, −5.2, and −3.7 kg in Participants 1–5, respectively, and between T2 and T3 by −15.0, −11.8, −9.3, −8.9, and −5.5 kg (Table 3, Figure 3). Body fat percentage decreased in four of five participants (in Participant 3 it increased from 41.3% to 42.4%), while skeletal muscle mass decreased numerically in all four tirzepatide-treated participants (by 2.6, 1.6, 3.2, and 3.6 kg in Participants 1–4, respectively, corresponding to 8.6%, 6.3%, 12.1%, and 10.3% of baseline values; Participant 5: −0.1 kg). Waist and hip circumferences decreased in each of the four participants receiving tirzepatide, although the magnitude of change varied. Participant 5, who received the non-pharmacological intervention alone, also showed anthropometric changes: WHtR decreased from 0.61 to 0.57 and WHR from 0.89 to 0.86 (Supplementary Material—Table S1). Lower-limb circumference changes varied by participant and measurement site (see individual case descriptions). Nevertheless, among all participants, reductions were generally observed at most sites, which was consistent with ultrasound measurements. Individual patients’ characteristics are presented in Table 2 and anthropometric ratios in Table 4, Table 5, Table 6, Table 7 and Table 8. Fasting glucose and HOMA-IR decreased in most cases, whereas lipid changes were mixed (Table 9). The two eligible participants who did not complete follow-up discontinued follow-up because they were unable to travel to the clinic for visits scheduled at predefined time points.

All four participants who started tirzepatide at T2 completed the planned 14–16-week dose-escalation schedule and remained on 7.5 mg once weekly until T3. No adverse events were reported. In particular, no nausea, constipation, or other gastrointestinal symptoms were reported, and no dose reductions, treatment interruptions, discontinuations, or serious adverse events were recorded.

### 3.1. Individual Case Descriptions

**Participant 1**

The first participant was a 30-year-old woman diagnosed with lipedema of the lower limbs (stage 2, type III) and upper limbs (stage 1, type IV) (Figure 4), which began with pubertal development and gradually worsened over the years and after pregnancy (G1 P1), 3 years before evaluation. The lowest body weight in adulthood was 54 kg and the highest 96.3 kg with BMI 31.8 kg/m^2^, at the time of evaluation. The patient had concomitant chronic venous insufficiency class C2 in the CEAP (Clinical–Etiology–Anatomy–Pathophysiology) classification, with a history of bilateral saphenectomy. In the past, she suffered from primary immune thrombocytopenia, due to which she underwent splenectomy in 2013. In addition, she suffered from depression, treated with venlafaxine at a dose of 187.5 mg per day. During the study period, the patient was not taking hormonal contraception or any other hormone therapy. At baseline (T1), circumferences were recorded at all seven segmental lower-limb points and at the waist and hip. SAT thickness was assessed by ultrasound (Table 4). During the initial 8–9-week diet-and-exercise-only phase (T1 to T2), waist circumference decreased modestly (86.0 to 85.0 cm) while several limb circumferences (cB, cB1, cC, cF, cG) showed small numerical increases, most pronounced at cF (+5.0 cm). The participant reported, on clinical interview, incomplete adherence to the prescribed dietary recommendations during this phase, which may account for the observed circumferential increases despite the intended energy deficit. By T3 (week 22–25), hip circumference decreased more than waist circumference (122.0 to 117.0 cm vs. 86.0 to 85.0 cm), resulting in a numerical increase in WHR (0.70 to 0.73) despite a stabilization of WHtR (0.49) (Supplementary Material—Table S1). Lower-limb circumferences decreased at all seven segmental points, with the largest reductions around the knee (cD, −6.0 cm; cE, −6.0 cm) (Table 4). The CEAP score remained unchanged. All eight SF-36 domains improved from T2 to T3, with the largest absolute gains in Emotional Role, Social Functioning, and Mental Health domains (Table 10). In contrast, pressure pain threshold improved at three of seven sites (cB, cD, cE), remained unchanged at one (cF), and decreased at three others (cB1, cC, cG), including a marked reduction at the proximal thigh (cG) despite a concurrent reduction in thigh circumference at that site—illustrating a dissociation between patient-reported pain relief and objectively measured mechanical tissue sensitivity (Table 11).

**Table 4:** The table shows the lower-limb circumferences and the adipose tissue thickness of Participant 1, measured with ultrasound. The data before (T1), after 8–9 weeks (T2), and after 22–25 weeks (T3) of intervention, as well as the variation from the baseline (Delta T3–T1), are reported. Circumference values are bilateral means ((right + left)/2); each limb value is the mean of two measurements.

	T1	T2	T3	Delta (T3–T1)
Lower-limb circumference (cm)				
cB	27.8	28.0	26.7	−1.1
cB1	41.3	43.0	40.0	−1.3
cC	49.0	51.0	46.8	−2.2
cD	46.0	43.0	40.0	−6.0
cE	55.0	54.0	49.0	−6.0
cF	68.0	73.0	66.5	−1.5
cG	75.8	76.0	71.1	−4.7
Thickness of the subcutaneous tissue (mm)				
Anterior thigh	31.6		26.8	−4.8
right/left leg	32.6		28.3	−4.3
Pre-tibial region	26.2		26.2	0.0
right/left leg	31.5		31.0	−0.5
Lateral calf	30.2		27.3	−2.9
right/left leg	27.8		26.6	−1.2
Medial supramalleolar	43.0		33.5	−9.5
right/left leg			34.4	−8.6

Measurement locations: cB/cB1 = distal calf (two sites); cC–cF = progressive locations along the lower leg; cG = proximal thigh. Anterior thigh: midpoint between the iliac crest and the lower patellar border; Pre-tibial region: midpoint between anterior tibial tuberosity and medial malleolus; Lateral calf: midpoint between lateral malleolus and fibular head; medial supramalleolar: 6 cm above the medial malleolus.

**Participant 2**

The second participant was a 45-year-old woman diagnosed with lipedema of the lower limbs (stage 2, type III), which began after pregnancies (G2 P2) (Figure 5). The lowest and highest body weights in adulthood were 48 and 92 kg, respectively, with a weight of 88.3 kg and a BMI of 32.4 kg/m^2^ at the time of our evaluation. The patient had concomitant chronic venous insufficiency class C2 in the CEAP classification, with no history of endovenous or surgical treatment. Duplex Doppler ultrasound revealed the great saphenous vein insufficiency with insufficient tributaries in the right limb. At T1, she was not taking any medications, including hormonal contraception or hormone therapy. At baseline (T1), circumferences were measured at seven segmental lower-limb points, as well as at the waist and hip. SAT thickness was assessed by ultrasound (Table 5). During the initial 8–9-week diet-and-exercise-only phase (T1 to T2), both waist and hip circumferences decreased (waist: 96.5 to 92.0 cm; hip: 120.0 to 117.0 cm). Limb circumferences decreased at six of seven segmental points, with only cB showing no changes. By T3 (week 22–25), waist and hip circumferences had decreased further (waist: 92.0 to 81.5 cm; hip: 117.0 to 108.0 cm), resulting in improvements in both waist-to-hip ratio (WHR: 0.80 to 0.75) and waist-to-height ratio (WHtR: 0.58 to 0.49) from T1 to T3 (Table S1). Lower-limb circumferences decreased at six of seven segmental points from T1 to T3, ranging from no change at cB to reductions of approximately 6–10% at the remaining six points. The largest relative reductions were observed at cD (−10.2%) and cE (−10.3%) (Table 4). The CEAP score remained unchanged. On SF-36, four domains improved from T2 to T3 (Physical Functioning, General Health, Vitality, Mental Health). Two domains remained unchanged at the ceiling score of 100 (Physical Role and Bodily Pain), while two domains decreased (Social Functioning, Emotional Role). Pressure pain threshold decreased at all seven measured sites from T2 to T3, indicating increased mechanical tissue sensitivity across the assessed lower-limb points (Table 11). This combination of favorable anthropometric changes, a uniform decline in pressure pain threshold, and a mixed quality-of-life response differs from the pattern observed in Participant 1. These findings highlight the inter-individual heterogeneity of treatment response in this case series. Rosuvastatin 5 mg daily was started during the observation period (April 2025), which should be considered a potential confounder when interpreting this participant’s lipid changes between T1 and T3.

**Table 5:** The table shows the lower-limb circumferences and the adipose tissue thickness of Participant 2, measured with ultrasound. The data before (T1), after 8–9 weeks (T2), and after 22–25 weeks (T3) of intervention, as well as the variation from the baseline (Delta T3–T1), are reported. Circumference values are bilateral means ((right + left)/2); each limb value is the mean of two measurements.

	T1	T2	T3	Delta (T3–T1)
Lower-limb circumference (cm)				
cB	25.8	25.8	25.8	0
cB1	33.8	32.0	31.5	−2.3
cC	43.0	42.0	40.3	−2.7
cD	41.8	40.0	37.5	−4.3
cE	48.5	46.0	43.5	−5.0
cF	58.5	58.3	54.8	−3.7
cG	69.5	67.5	62.8	−6.7
Thickness of the subcutaneous tissue (mm)				
Anterior thigh	27.5		19.7	−7.8
right/left leg	27.9		19.1	−8.8
Pre-tibial region	23.0		19.4	−3.6
right/left leg	22.0		19.8	−2.2
Lateral calf	19.6		15.7	−3.9
right/left leg	19.5		16.7	−2.8
Medial supramalleolar	25.9		22.6	−3.3
right/left leg			20.9	−1.1

Measurement locations: cB/cB1 = distal calf (two sites); cC–cF = progressive locations along the lower leg; cG = proximal thigh. Anterior thigh: midpoint between the iliac crest and the lower patellar border; Pre-tibial region: midpoint between anterior tibial tuberosity and medial malleolus; Lateral calf: midpoint between lateral malleolus and fibular head; medial supramalleolar: 6 cm above the medial malleolus.

**Participant 3**

The third participant was a 36-year-old woman diagnosed with lipedema of the lower limbs (stage 1, type II), which began with pubertal development (Figure 6). There was no history of previous pregnancies. The lowest body weight in adulthood was 48 kg and the highest was 82.8 kg, with a BMI of 30.4 kg/m^2^ at the time of our evaluation. The patient reported a feeling of heaviness in the lower limbs with no visible changes upon physical examination and Doppler ultrasound (CEAP C0). Family history was notable for lipedema on the maternal side and diabetes on the paternal side, including a paternal grandparent. At T1, she was not taking any medications. At baseline (T1), circumferences were measured at seven segmental lower-limb points as well as at the waist and hip. SAT thickness was assessed by ultrasound (Table 6). During the initial 8–9-week diet-and-exercise-only phase (T1 to T2), waist circumference decreased modestly (82.0 to 80.0 cm) while hip circumference showed a smaller reduction (122.0 to 119.0 cm); WHR remained essentially unchanged during this phase (0.67). By T3 (week 22–25), waist and hip circumferences had decreased further (waist: 80.0 to 75.0 cm; hip: 119.0 to 114.5 cm), resulting in improvements in both WHR (0.67 to 0.66) and WHtR (0.50 to 0.45) from T1 to T3 (Table S1). Lower-limb circumferences decreased at six of seven segmental points from T1 to T3, ranging from 0.0% at cE to −8.3% at cD (Table 6). The CEAP score remained unchanged. On SF-36, this participant had markedly impaired baseline (T2) scores in Physical Role (0/100) and Emotional Role (0/100), together with a low Bodily Pain score (13/100); all eight domains improved from T2 to T3, with the largest absolute gains in Bodily Pain, Emotional Role, Physical Role, and Social Functioning. Pressure pain threshold improved at the three most proximal sites (cE, cF, cG), with the largest improvement at cG (2.38 to 4.63 kgf/cm^2^), but decreased at the four more distal sites (cB, cB1, cC, cD; Table 11). Body weight was 82.8 kg at T1, 84.4 kg at T2, and 75.1 kg at T3.

**Table 6:** The table shows the lower-limb circumferences and the adipose tissue thickness of Participant 3, measured with ultrasound. The data before (T1), after 8–9 weeks (T2), and after 22–25 weeks (T3) of intervention, as well as the variation from the baseline (Delta T3–T1), are reported. Circumference values are bilateral means ((right + left)/2); each limb value is the mean of two measurements.

	T1	T2	T3	Delta (T3–T1)
Lower-limb circumference (cm)				
cB	24.5	24.0	23.8	−0.7
cB1	33.8	33.5	32.1	−1.7
cC	43.9	44.5	42.3	−1.6
cD	44.5	44.5	40.8	−3.7
cE	47.3	48.5	47.3	0.0
cF	68.8	68.5	67.8	−1.0
cG	75.8	75.5	72.8	−3.0
Thickness of the subcutaneous tissue (mm)				
Anterior thigh	35.3		30.6	−4.7
right/left leg	36.7		31.5	−5.2
Pre-tibial region	15.6		11.3	−4.3
right/left leg	17.8		15.3	−2.5
Lateral calf	17.7		17.7	0.0
right/left leg	19.5		19.0	−0.5
Medial supramalleolar	21.6		17.4	−4.2
right/left leg			17.9	0.0

Measurement locations: cB/cB1 = distal calf (two sites); cC–cF = progressive locations along the lower leg; cG = proximal thigh. Anterior thigh: midpoint between the iliac crest and the lower patellar border; Pre-tibial region: midpoint between anterior tibial tuberosity and medial malleolus; Lateral calf: midpoint between lateral malleolus and fibular head; medial supramalleolar: 6 cm above the medial malleolus.

**Participant 4**

The fourth participant was a 26-year-old woman diagnosed with lipedema of the lower limbs (stage 2, type II), which began with pubertal development and worsened after progressive weight gain (Figure 7). The patient has no history of previous pregnancies. The lowest and highest body weights in adulthood were 75 and 125 kg, respectively, with a weight of 116.1 kg and BMI of 38.3 kg/m^2^ at the time of our evaluation. On physical examination, small, isolated telangiectasias were observed, which correspond to class C1 in the CEAP classification. Duplex Doppler ultrasound revealed no reflux in the main superficial venous trunks and tributaries. She suffered from autoimmune hypothyroidism, treated with levothyroxine at a dose of 50/75 ug per day, with no use of hormonal contraception or other hormonal therapy during the study flow. At baseline (T1), circumferences were measured at seven segmental lower-limb points as well as at the waist and hip. SAT thickness was assessed by ultrasound (Table 7). During the initial 8–9-week diet-and-exercise-only phase (T1 to T2), waist circumference decreased (106.0 to 101.0 cm) while hip circumference increased slightly (127.0 to 128.0 cm), resulting in an improvement in WHR during this phase (0.83 to 0.79). By T3 (week 22–25), waist and hip circumferences had both decreased further (waist: 101.0 to 98.0 cm; hip: 128.0 to 121.5 cm); WHtR improved consistently across both phases (0.61 to 0.58 to 0.56), although it remained above the 0.5 cardiometabolic risk threshold throughout the observation period. WHR showed a smaller net improvement from T1 to T3 (0.83 to 0.81), reflecting a partial reversal between T2 and T3 (0.79 to 0.81) as hip circumference decreased more than waist circumference proportionally during the tirzepatide phase (Table S1). Lower-limb circumferences showed modest reductions at six of seven segmental points from T1 to T3 (range: 0.0% to −5.3%), a narrower range of change than observed in the previously described participants (Table 7). The CEAP score remained unchanged. On SF-36, five of eight domains improved from T2 to T3 (Physical Role, Bodily Pain, Vitality, Social Functioning, Emotional Role); Physical Functioning remained unchanged, while General Health and Mental Health both decreased, with no relevant clinical history accounting for this decline identified on interview (Table 10). Pressure pain threshold improved at six of seven measured sites and remained unchanged at one (cF) (Table 11). This was the only fully consistent (non-mixed) directional pattern in pressure pain threshold among Participants 1–4. Baseline PPT values in this participant were also markedly lower than in previously described participants (range: 0.88–1.88 kgf/cm^2^ vs. 2.00–6.00 kgf/cm^2^), consistent with greater baseline mechanical tissue sensitivity in the participant with the highest baseline body mass index in the cohort.

**Table 7:** The table shows the lower-limb circumferences and the adipose tissue thickness of Participant 4, measured with ultrasound. The data before (T1), after 8–9 weeks (T2), and after 22–25 weeks (T3) of intervention, as well as the variation from the baseline (Delta T3–T1), are reported. Circumference values are bilateral means ((right + left)/2); each limb value is the mean of two measurements.

	T1	T2	T3	Delta (T3–T1)
Lower-limb circumference (cm)				
cB	25.5	26.0	25.4	−0.1
cB1	32.8	33.0	32.8	0.0
cC	45.8	47.0	45.5	−0.3
cD	43.5	46.0	42.3	−1.2
cE	53.0	52.0	51.0	−2.0
cF	69.8	71.0	69.3	−0.5
cG	80.8	80.0	76.5	−4.3
Thickness of the subcutaneous tissue (mm)				
Anterior thigh	26.6		24.9	−1.7
right/left leg	35.7		23.5	−12.2
Pre-tibial region	25.3		24.5	−0.8
right/left leg	25.6		24.6	−1.0
Lateral calf	12.8		12.8	0.0
right/left leg	12.8		12.2	−0.6
Medial supramalleolar	19.3		15.9	−3.4
right/left leg			17.0	

Measurement locations: cB/cB1 = distal calf (two sites); cC–cF = progressive locations along the lower leg; cG = proximal thigh. Anterior thigh: midpoint between the iliac crest and the lower patellar border; Pre-tibial region: midpoint between anterior tibial tuberosity and medial malleolus; Lateral calf: midpoint between lateral malleolus and fibular head; medial supramalleolar: 6 cm above the medial malleolus.

**Participant 5**

The fifth participant was a 60-year-old woman diagnosed with lipedema of the lower limbs (stage 2, type II), which began with pubertal development and gradually worsened over the years and after pregnancies (G2 P2) (Figure 8). The lowest and highest body weight in adulthood were 56 and 96 kg, respectively, with a weight of 76.4 kg and a BMI of 32.2 kg/m^2^ at the time of our evaluation. Upon physical examination, telangiectasias and reticular veins were observed, which correspond to class C1 in the CEAP classification. Duplex Doppler ultrasound revealed no reflux in the main superficial venous trunks and tributaries. She suffered from arterial hypertension and combined hyperlipidemia, treated with a fixed-dose combination of perindopril/amlodipine/indapamide (Triplixam 10/2.5/10 mg); in April 2025, during the observation period, antihypertensive treatment was changed to ramipril 10 mg once daily (Tritace) plus the diuretic indapamide 2.5 mg once daily (Indapen) and transdermal hormone replacement therapy (patch).

Participant 5 declined pharmacological treatment and maintained the diet-and-exercise-only intervention throughout the 22–25-week observation period, without receiving tirzepatide at any time. Central circumferences decreased over the observation period. Waist circumference remained stable during the initial 8–9-week diet-and-exercise-only phase (94.0 cm) and then decreased by T3 (94.0 to 88.5 cm). Hip circumference decreased steadily throughout (105.5 to 105.0 to 102.5 cm). Waist-to-hip ratio (WHR) showed a small improvement from T1 to T3 (0.89 to 0.86), with a slight increase during the initial phase (0.90). Waist-to-height ratio (WHtR) decreased from 0.61 to 0.57 but remained above the 0.5 cardiometabolic risk threshold throughout the observation period (Table S1). Lower-limb circumferences decreased at all seven segmental points from T1 to T3 (range: −0.9% to −9.2%), with a biphasic pattern at cE, characterized by a reduction from T1 to T2 and a partial increase from T2 to T3 (net −3.9%) (Table 8). The CEAP score remained unchanged. On SF-36, six domains improved or remained stable (Physical Functioning, Bodily Pain, General Health; unchanged: Vitality, Social Functioning, Mental Health). In contrast, Physical Role and Emotional Role both declined to the floor score (Table 10), despite continued anthropometric improvement during the same period. The participant reported on the study questionnaire uncertainty in interpreting certain SF-36 items, despite access to standard instructions, and suggested that the instrument might be better adapted to reflect the specific experiences of individuals with lipedema. This observation raises the possibility that the marked decline in these two domains may partly reflect difficulty with item interpretation rather than a genuine deterioration in role functioning. Pressure pain threshold improved at three of seven measured sites (cD, cF, cG), remained unchanged at two (cB, cE), and decreased at two (cB1, cC) (Table 11). Clinically, lower-limb edema decreased over the course of the intervention.

**Table 8:** The table shows the lower-limb circumferences and the adipose tissue thickness of Participant 5, measured with ultrasound. The data before (T1), after 8–9 weeks (T2), and after 22–25 weeks (T3) of intervention, as well as the variation from the baseline (Delta T3–T1), are reported. Circumference values are bilateral means ((right + left)/2); each limb value is the mean of two measurements.

	T1	T2	T3	Delta (T3–T1)
Lower-limb circumference (cm)				
cB	25.5	25.0	25.0	−0.5
cB1	29.0	29.0	28.8	−0.2
cC	41.0	41.0	39.8	−1.2
cD	41.3	40.0	37.5	−3.8
cE	45.0	41.0	43.3	−1.7
cF	57.0	55.0	51.8	−5.2
cG	62.5	63.0	57.3	−5.2
Thickness of the subcutaneous tissue (mm)				
Anterior thigh	25.1		17.6	−7.5
right/left leg	25.9		18.5	−7.4
Pre-tibial region	10.4		10.3	−0.1
right/left leg	10.9		10.9	0.0
Lateral calf	18.9		15.8	−3.1
right/left leg	19.1		17.3	−1.8
Medial supramalleolar	20.9		20.7	−0.2
right/left leg			20.3	−2.0

Measurement locations: cB/cB1 = distal calf (two sites); cC–cF = progressive locations along the lower leg; cG = proximal thigh. Anterior thigh: midpoint between the iliac crest and the lower patellar border; Pre-tibial region: midpoint between anterior tibial tuberosity and medial malleolus; Lateral calf: midpoint between lateral malleolus and fibular head; medial supramalleolar: 6 cm above the medial malleolus.

### 3.2. Biochemical Characteristics

Individual values for hs-CRP, complete blood count indices, alanine aminotransferase, thyroid-stimulating hormone, creatinine, estimated glomerular filtration rate (CKD-EPI), 25-OH vitamin D, and urinary albumin-to-creatinine ratio at T1 and T3 are reported for each participant in Table 9. Several values require comment. Participant 5 had markedly elevated total cholesterol (325.7 and 313.3 mg/dL at T1 and T3) and LDL-C (253.4 and 245.6 mg/dL), consistent with severe hypercholesterolemia (LDL-C ≥190 mg/dL); familial hypercholesterolemia cannot be excluded. Participant 5 also had suppressed TSH at both time points (0.197 and 0.134 µIU/mL); this finding was present before the intervention, but subclinical hyperthyroidism may be relevant to her cardiovascular risk and body composition. In Participant 1, hs-CRP increased from 2.10 to 4.70 mg/L; a single hs-CRP measurement can be affected by intercurrent infection or other acute conditions, and this change should not be interpreted as a treatment effect. hs-CRP was also above 3 mg/L in Participant 5 at T3 (4.72 mg/L; T1 value not available). Participant 2 had vitamin D deficiency at baseline (13.6 ng/mL), which resolved with supplementation (45.5 ng/mL at T3). Blood count, ALT, creatinine, and eGFR values remained within reference ranges. Fasting glucose and HOMA-IR decreased in most participants. Lipid changes were variable: HDL-C decreased in Participants 1–4, LDL-C increased in Participants 1, 3, and 4, and LDL-C and total cholesterol decreased in Participant 2, who started rosuvastatin during the observation period (Table 9).

**Table 9:** Biochemical characteristics at T1 (week 0, before the 8–9-week diet-and-exercise-only phase) and T3 (week 22–25, after 14–16 weeks of tirzepatide exposure in Participants 1–4). Participant 5 did not receive tirzepatide.

	8–9-Week Diet/Exercise Phase, Then Tirzepatide (Weeks 8–9 to 22–25)				Diet and Exercise Throughout; No Tirzepatide
Characteristic	Participant 1	Participant 2	Participant 3	Participant 4	Participant 5
Fasting glucose (mg/dL) T1/T3					
	87	117	92	85	91
	82	77	84	81.9	83
Fasting insulin (mIU/l) T1/T3					
	4.6	6.8	13.0	17.9	16.8
	3.0	6.5	13.4	15.2	19.3
HOMA-IR T1/T3					
	0.99	1.96	2.95	3.76	3.78
	0.61	1.24	2.78	3.07	3.97
TC (mg/dL) T1/T3					
	174	184	203	198	325.7
	176	137	193	184	313.3
LDL-C (mg/dL) T1/T3					
	107	94	121	125	253.4
	117	61	123	130	245.6
HDL-C (mg/dL) T1/T3					
	59	79	65	58	40.2
	49	67	58	53.8	47.3
TG (mg/dL) T1/T3					
	35	62	97	82	163.4
	48	36	66	96.5	113.9
hs-CRP (mg/L) T1/T3					
	2.100	ND	1.250	0.750	ND
	4.700	1.290	0.700	0.470	4.720
Hemoglobin (g/dL) T1/T3					
	12.8	13.7	14.7	15.1	13.9
	13.4	13.7	14.6	15.7	13.8
MCV (fL) T1/T3					
	88.7	93.3	89.5	88.3	84.1
	88.7	91.0	87.2	87.8	86.8
Hematocrit (%) T1/T3					
	39.9	41.5	44.1	45.2	41.4
	40.8	40.5	41.7	47.4	42.6
ALT (U/L) T1/T3					
	19.0	22.0	13.0	33.0	10.0
	10.0	17.0	14.0	22.3	14.0
TSH (µIU/mL) T1/T3					
	1.780	1.472	2.450	2.510	0.197
	1.200	0.997	2.100	2.440	0.134
Creatinine (mg/dL) T1/T3					
	0.79	0.62	0.71	0.66	0.88
	0.91	0.65	0.79	0.76	0.64
eGFR (CKD-EPI, mL/min/1.73m^2^) T1/T3					
	100.6	108.4	109.1	119.7	75.2
	87.0	106.8	95.9	106.3	96.6
25-OH vitamin D3 (ng/mL) T1/T3					
	44.8	13.6	35.3	23.1	52.6
	54.3	45.5	41.7	30.5	39.6
Urinary albumin-to-creatinine ratio, ACR (mg/g) T1/T3					
	0.00	ND	0.00	0.00	ND
	2.60	4.52	0.00	ND	0.00

HOMA-IR—Homeostasis Model Assessment of Insulin Resistance, TC—total cholesterol, LDL—low-density lipoprotein, HDL—high-density lipoprotein, TG—triglycerides; hs-CRP—high-sensitivity C-reactive protein, HGB—hemoglobin, MCV—mean corpuscular volume, HCT—hematocrit, ALT—alanine aminotransferase, TSH—thyroid-stimulating hormone, eGFR—estimated glomerular filtration rate (CKD-EPI equation), ACR—urinary albumin-to-creatinine ratio, ND—not determined (result not available for that timepoint).

### 3.3. Quality of Life and Bodily Pain

SF-36 domain scores (assessed only at T2 and T3) generally increased among participants 1–4, with the largest average absolute changes observed for Bodily Pain and Emotional Role. Participant 5 showed improvement in Bodily Pain, Physical Functioning and General Health, whereas Physical Role and Emotional Role decreased to 0 (Table 10). Because the SF-36 was not administered at T1, these data reflect only the period from T2 to T3 and not the full course; in Participants 1–4, they cannot be attributed to tirzepatide alone.

**Table 10:** SF-36 Quality of Life Assessment.

Characteristic	Participant 1	Participant 2	Participant 3	Participant 4	Participant 5
Physical Functioning					
T2/T3	80	95	70	75	65
	90	100	95	75	70
Physical Role					
T2/T3	75	100	0	0	25
	100	100	50	50	0
Bodily Pain					
T2/T3	23	100	13	55	35
	68	100	90	78	45
General Health					
T2/T3	60	80	75	45	70
	90	100	90	40	80
Vitality					
T2/T3	45	50	40	50	80
	50	80	70	60	80
Social Functioning					
T2/T3	13	100	38	63	50
	75	38	88	75	50
Emotional Role					
T2/T3	38	100	0	33	67
	100	80	67	67	0
Mental Health					
T2/T3	40	72	32	80	68
	88	80	80	56	76

Values are individual data for each of the five participants (not aggregated). T2 = week 8–9, immediately before tirzepatide initiation in Participants 1–4; T3 = week 22–25, after 14–16 weeks of tirzepatide exposure in Participants 1–4. All SF-36 domains are scored from 0 to 100, with higher scores indicating better quality of life. The SF-36 was not administered at T1; changes during the initial 8–9-week diet-and-exercise phase were therefore not assessed. Participant 5 continued diet and physical activity without tirzepatide.

Pressure pain threshold responses varied among participants (Table 11). No consistent directional pattern could be discerned in most locations. Individual changes ranged from marked improvement to marked worsening in the same anatomical location, depending on the participant. These results contrasted with the improvement in bodily pain reported by patients on the SF-36 (Table 10).

**Table 11:** Pressure pain threshold (algometry).

Characteristic	Participant 1	Participant 2	Participant 3	Participant 4	Participant 5
cB, Pre (T2)	3.00	4.88	5.00	1.63	2.38
cB, Post (T3)	4.25	3.63	4.75	2.00	2.38
cB1, Pre (T2)	2.50	6.00	5.00	1.88	2.75
cB1, Post (T3)	1.88	4.63	4.75	2.13	2.50
cC, Pre (T2)	2.50	5.88	6.00	1.88	3.38
cC, Post (T3)	1.88	4.38	3.88	2.00	3.00
cD, Pre (T2)	2.50	6.00	5.25	1.50	1.50
cD, Post (T3)	2.75	4.38	3.88	1.63	2.25
cE, Pre (T2)	3.13	4.62	3.75	1.05	3.50
cE, Post (T3)	3.28	4.40	4.13	1.38	3.50
cF, Pre (T2)	2.00	6.00	3.25	1.13	1.63
cF, Post (T3)	2.00	3.88	3.63	1.13	1.88
cG, Pre (T2)	2.63	6.00	2.38	0.88	1.75
cG, Post (T3)	1.75	3.50	4.63	1.00	2.75

Values are in kgf/cm^2^. Pre = T2 (week 8–9, immediately before tirzepatide initiation); Post = T3 (week 22–25, after 14–16 weeks of tirzepatide exposure in Participants 1–4). Higher pressure pain threshold values indicate lower pain sensitivity. Participant 5 continued diet and physical activity without tirzepatide. Responses among Participants 1–4 were heterogeneous and should be interpreted descriptively.

### 3.4. Follow-Up After T3 (Exploratory)

The following observations were obtained after T3 at non-standardized time points (Table 12). The most recent contact with all participants took place on 14 September 2026 by telephone and e-mail. Observations recorded after ethics approval and verbal consent to follow-up in 2026 constitute the prospective follow-up component of the study; the observation of 18 December 2025 was obtained from the medical record. Because no standardized assessments were scheduled for this period, these observations were not part of the main outcomes, are reported as exploratory, and cannot be used to assess the durability of the changes described above.

## 4. Discussion

This ambispective pilot case series describes five women with lipedema and excess body weight who completed a 22–25-week clinical course. All participants first completed 8–9 weeks of diet and physical activity alone. At T2, four participants received tirzepatide for the remaining 14–16 weeks, whereas one continued the non-pharmacological intervention alone. The participant who did not receive tirzepatide was not a control and had a distinct baseline metabolic profile. Because tirzepatide was given together with diet, exercise, and supplementation, without randomization or a control group, the observations discussed below are descriptive and do not permit any inference about the effect of tirzepatide or about a lipedema-specific effect.

Participant 5 cannot serve as a control for several reasons. First, she was not allocated by randomization; she declined pharmacotherapy, and this self-selection may be associated with differences in motivation, treatment expectations, and health behaviors. Second, her baseline characteristics differed substantially from those of Participants 1–4: she was the oldest participant (60 vs. 26–45 years), was receiving transdermal hormone replacement therapy, had markedly elevated LDL-C and suppressed TSH, and her antihypertensive treatment was changed during the observation period. Third, a single individual provides no estimate of between-person variability, so any difference between her course and that of the treated participants could reflect chance, baseline differences, or concurrent treatments rather than the absence of tirzepatide. Her data are therefore reported as an individual case description only, and no between-group comparison is made or implied.

The observations are in line with current recommendations supporting weight management, physical activity, and anti-inflammatory dietary patterns in lipedema [2,5]. Previous studies have reported reductions in pain and improvements in quality of life with low-carbohydrate or ketogenic diets [12,26,27]. In a larger comparative study of 113 women, an LCHF diet was associated with significant reductions in body weight, body fat mass, and leg volume, together with significantly reduced pain, in women with lipedema [28]. The deterioration observed in the participant who received only the non-pharmacological intervention warrants explicit discussion, as this is the only case in which the non-pharmacological intervention was continued without tirzepatide. This participant experienced a decline in the Physical Role (25 to 0) and Emotional Role (67 to 0) domains of the SF-36, between T2 and T3, reaching the minimum score, despite improvements in body weight and both central and lower-limb circumferences, from T1 to T3. As noted in the Limitations, the participant also reported difficulty interpreting certain SF-36 items, which may partially explain the marked decline in these two domains rather than indicating a true deterioration in role functioning. In this single case, anthropometric improvements were not accompanied by consistent improvements across all quality-of-life domains; no general conclusion can be drawn from one participant.

Weight loss has also been described in a small case series of exenatide in lipedema [14], and tirzepatide reduces body weight and modifies fat distribution in other populations [29]; however, the present design does not allow the changes observed here to be attributed to tirzepatide. In the present series, circumference reductions were generally greater in proximal than distal segments, a pattern that warrants comment in relation to the classical description of lipedemic tissue as resistant to conventional weight loss [5,11]. The smallest reductions were observed at the most distal segmental points (cB, cB1), corresponding to the ankle and distal calf, where the disproportionate, symmetrical fat accumulation characteristic of lipedema is most classically described [1,2]. Reductions were also observed at these distal sites—both in the present series and in a larger comparative study, in which ankle circumference decreased more in women with lipedema than in overweight/obese controls without lipedema following a similar low-carbohydrate dietary intervention [28]. Given the concurrent weight loss, these observations cannot be taken as evidence that any component of the intervention affected lipedemic tissue specifically. In the present series, the gradient itself was not fully monotonic (the greatest reduction was observed at cD, an intermediate site), and the absence of tissue-composition imaging in the present study—which assessed only subcutaneous adipose tissue thickness by ultrasound—precludes any definitive conclusion about differential depot-specific responsiveness. Whether lipedemic depots respond differently from other adipose tissue should be examined in controlled studies using site-specific imaging.

However, reductions in body weight, limb circumference, and SAT thickness are expected with substantial weight loss of any cause and do not by themselves demonstrate modification of pathological lipedemic tissue. Distinguishing a lipedema-specific effect from the general consequences of weight loss would require, for example, comparison of proportional changes in affected limb regions with those in unaffected regions, assessment of tissue characteristics such as fibrosis, edema, or echogenicity, and a comparator group of women with obesity without lipedema. None of these was available in the present series. The changes reported here should therefore be interpreted as changes accompanying weight loss in women with lipedema, not as evidence of a lipedema-specific effect of the intervention or of tirzepatide.

Skeletal muscle mass decreased numerically in all four tirzepatide-treated participants (by 1.6–3.6 kg, i.e., 6.3–12.1% of baseline values) and was essentially unchanged in Participant 5 (−0.1 kg). Between T2 and T3, skeletal muscle mass decreased by 3.2, 1.8, 3.4, and 2.3 kg in Participants 1–4, respectively, corresponding to approximately 21%, 15%, 37%, and 26% of the body weight lost during that phase (Participant 5: −0.7 kg), whereas it changed little between T1 and T2 (from −1.3 to +0.6 kg). Some loss of lean tissue is expected during substantial weight reduction. In the dual-energy X-ray absorptiometry (DXA) substudy of SURMOUNT-1, approximately one quarter of the weight lost with tirzepatide was lean mass, a proportion similar to that observed with placebo plus lifestyle intervention [30]; in STEP 1, lean body mass accounted for approximately 38% of the weight lost with semaglutide [31,32]. DXA-derived lean mass is not equivalent to skeletal muscle mass estimated by bioelectrical impedance; nevertheless, the decreases observed here appear broadly within the range reported in these trials, although the contribution of tirzepatide cannot be separated from that of the concurrent diet and exercise. Several factors may have contributed. First, bioelectrical impedance estimates depend on body water and shifts in hydration, glycogen stores, and extracellular fluid during a low-carbohydrate diet, and rapid weight loss may lead to overestimation of muscle loss. Second, protein intake was planned at 16–21% of energy, corresponding to approximately 0.8–1.4 g/kg actual body weight per day, which in most participants is below the 1.2–1.6 g/kg/day suggested during active weight reduction with GLP-1-based therapy [32]; reduced appetite during tirzepatide treatment may have further lowered actual intake. Third, resistance exercise was recommended at least twice weekly, whereas at least three sessions per week are recommended to help preserve muscle mass during such therapy [32], and the diaries recorded two sessions per week. Because muscle strength and physical function were not measured, the functional relevance of these changes cannot be determined. Adequate protein intake, supervised resistance training, DXA-based body composition, and assessment of muscle function should be included in future studies of incretin-based therapy in lipedema.

These findings should be interpreted in the context of a recent narrative review that proposes tirzepatide as a potential disease-modifying therapy for lipedema, acting through combined effects on metabolic dysfunction, chronic inflammation, and tissue fibrosis [15]. This study did not include direct assessment of tissue fibrosis or a comprehensive panel of inflammatory biomarkers. Furthermore, the heterogeneous and non-monotonic patterns of circumferential and pressure-pain-threshold changes observed do not permit confirmation or refutation of the specific mechanical pathways proposed by Viana et al. Direct testing of this hypothesis, such as through paired tissue biopsy or imaging-based fibrosis markers before and after incretin-based therapy, constitutes an important direction for future controlled studies.

Patient-reported bodily pain improved, but algometry results were inconsistent. Both outcomes were assessed only at T2 and T3. Pain in lipedema is multifactorial and may be influenced by mechanical loading, mobility, metabolic factors, and central pain processing [21,33,34,35,36,37]. The discordance between SF-36 and local pressure pain thresholds supports the use of multidimensional outcomes, including anthropometry, imaging, function, and patient-reported measures [38,39,40,41,42].

### Limitations

The principal limitations of this study are the very small sample size, the lack of homogeneity between participants, the non-randomized design, and the absence of a comparable control group; the participant who did not receive tirzepatide was not a control and cannot support between-group inference. Recruitment through social media may have introduced selection bias toward a highly motivated, disease-aware population. All participants completed the same initial 8–9-week diet-and-exercise-only phase, but only four subsequently received tirzepatide for 14–16 weeks, and the multimodal design precludes attribution of the observed changes specifically to tirzepatide. This limitation applies to all outcomes reported in the Results, including body composition, circumferences, ultrasound, laboratory, pain, and quality-of-life measures. Because the main outcomes (T1–T3) were collected during routine clinical care and analyzed retrospectively rather than under a research protocol, the standardization and completeness of some assessments may have been limited, although all assessments were scheduled at fixed time points (T1, T2, and T3) and performed with the same equipment and procedures.

Adherence to the dietary and physical-activity plan was monitored through video consultations and participant-completed diaries rather than objective methods such as 24 h dietary recalls, ketonaemia/ketonuria testing, or accelerometry; one participant reported only partial adherence at post-study follow-up. Bioelectrical impedance measurements were standardized for fasting status and bladder voiding but not for time of day (measurement times 09:09–13:21) or menstrual cycle phase, which may have influenced hydration-sensitive parameters in this population. Ultrasound was performed at all protocol sites, but inter- and intra-rater reliability were not assessed, the sonographer was not blinded to time point, and ultrasound was not repeated at T2; magnetic resonance imaging was not available, although no current guideline recommends it as a routine diagnostic tool in lipedema. Lower-limb circumferences are reported as bilateral means ((right + left)/2), so right- and left-limb values were not analyzed separately, and potential interlimb asymmetry could not be assessed. Pressure pain threshold results were heterogeneous, and the study was not powered to evaluate safety. Pressure pain threshold was assessed only at T2 and T3, so the diet-and-exercise phase could not be evaluated with this outcome. Skeletal muscle mass was estimated by bioelectrical impedance rather than DXA, and muscle strength and physical function were not assessed. Individual laboratory values (hs-CRP, complete blood count, liver enzymes, creatinine, eGFR, thyroid-stimulating hormone, 25-OH vitamin D, and urinary albumin-to-creatinine ratio) are reported for all participants in Table 9, but co-interventions such as compression therapy, manual lymphatic drainage, and intermittent pneumatic compression were not systematically recorded. Lipid-lowering medication was identified in one participant (Participant 2, rosuvastatin, started during the observation period) and should be considered a confounder of her lipid results; use of such medication was not systematically ascertained in the remaining participants. Menopausal and hormonal status were not documented for all participants, although Participant 5’s use of transdermal hormone replacement therapy is reported. Tirzepatide is approved for obesity but not for lipedema, and its use here should be regarded as off-label with respect to the lipedema indication; tirzepatide, vitamin D3, and the omega-3-6-9 supplement were purchased by the participants themselves. Participant 5’s markedly elevated total and LDL cholesterol and suppressed TSH are described in Section 3.2 but were not further characterized within the study.

The prospective follow-up after T3 did not include standardized assessments. Informal follow-up contact, obtained at variable and non-standardized time points, is reported separately in Section 3.4; these observations cannot substitute for a systematic durability assessment. In addition, one participant found certain SF-36 items difficult to interpret in the context of lipedema, highlighting a broader limitation of using a generic, non-disease-specific quality-of-life instrument in this population. The SF-36 was administered only at T2 and T3; therefore, it does not capture changes in quality of life during the initial 8–9-week diet-and-exercise phase (T1–T2), and the full multimodal course was not assessed using this outcome. Changes from T2 to T3 cannot be attributed to tirzepatide, given the lack of a comparator and the continued diet and exercise throughout.

Despite these limitations, this case series provides descriptive, hypothesis-generating observations that may inform the design of controlled trials of incretin-based therapy in lipedema.

## 5. Conclusions

In this pilot case series, a 22–25-week clinical course comprising 8–9 weeks of diet and physical activity alone followed by 14–16 weeks of tirzepatide in four participants was accompanied by reductions in body weight in all participants, in body fat percentage in four of five participants, and in most lower-limb circumference and SAT thickness measurements, together with changes in selected metabolic measures and quality of life. Pressure pain threshold responses were inconsistent, lipid changes were mixed, and skeletal muscle mass decreased numerically in all four tirzepatide-treated participants. Considerable inter-individual heterogeneity was observed across nearly all outcome domains, underscoring the value of individual-level reporting in this small case series. Because all participants received concurrent dietary and exercise interventions, these preliminary observations cannot be attributed to tirzepatide; they do not establish a lipedema-specific or causal effect, nor the safety of tirzepatide in this population, and require confirmation in larger controlled studies.

## Figures and Tables

**Figure 1:**
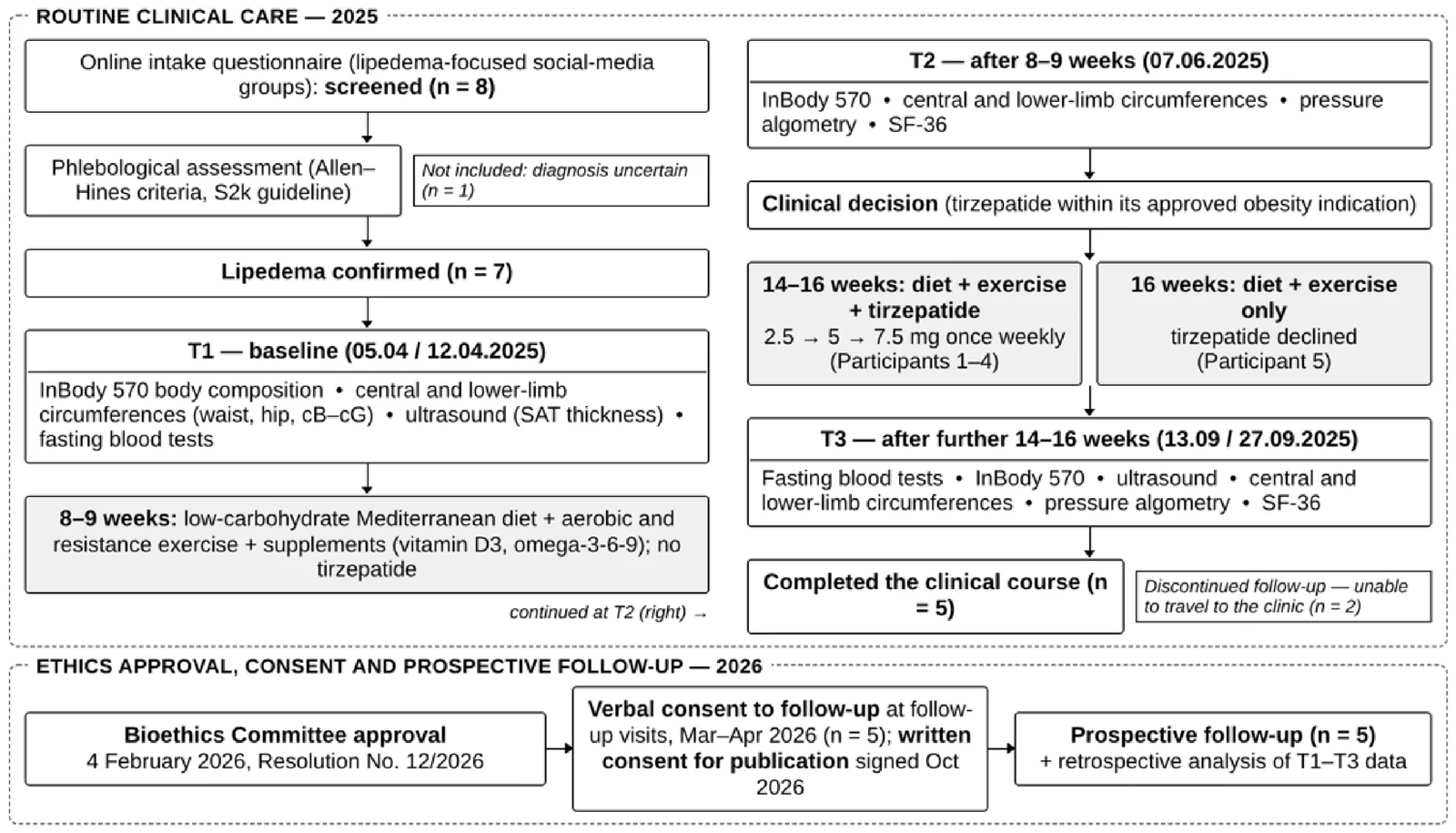
Participant flow and timeline: routine clinical care in 2025 (T1–T3; retrospective component) and ethics approval, verbal consent to follow-up, and prospective follow-up in 2026.

**Figure 2:**
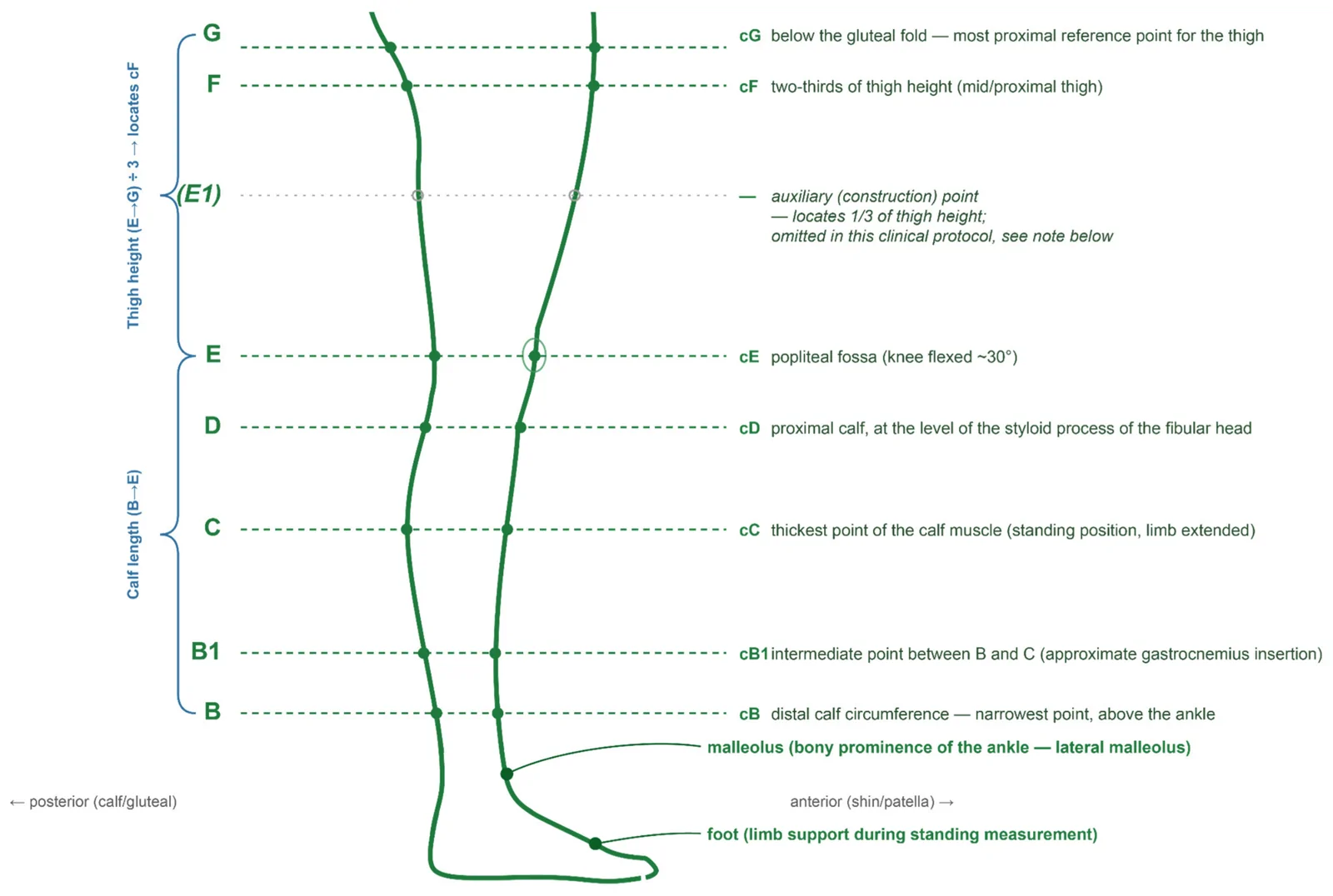
Schematic image of measurement points according to the segmental measurement technique.

**Figure 3:**
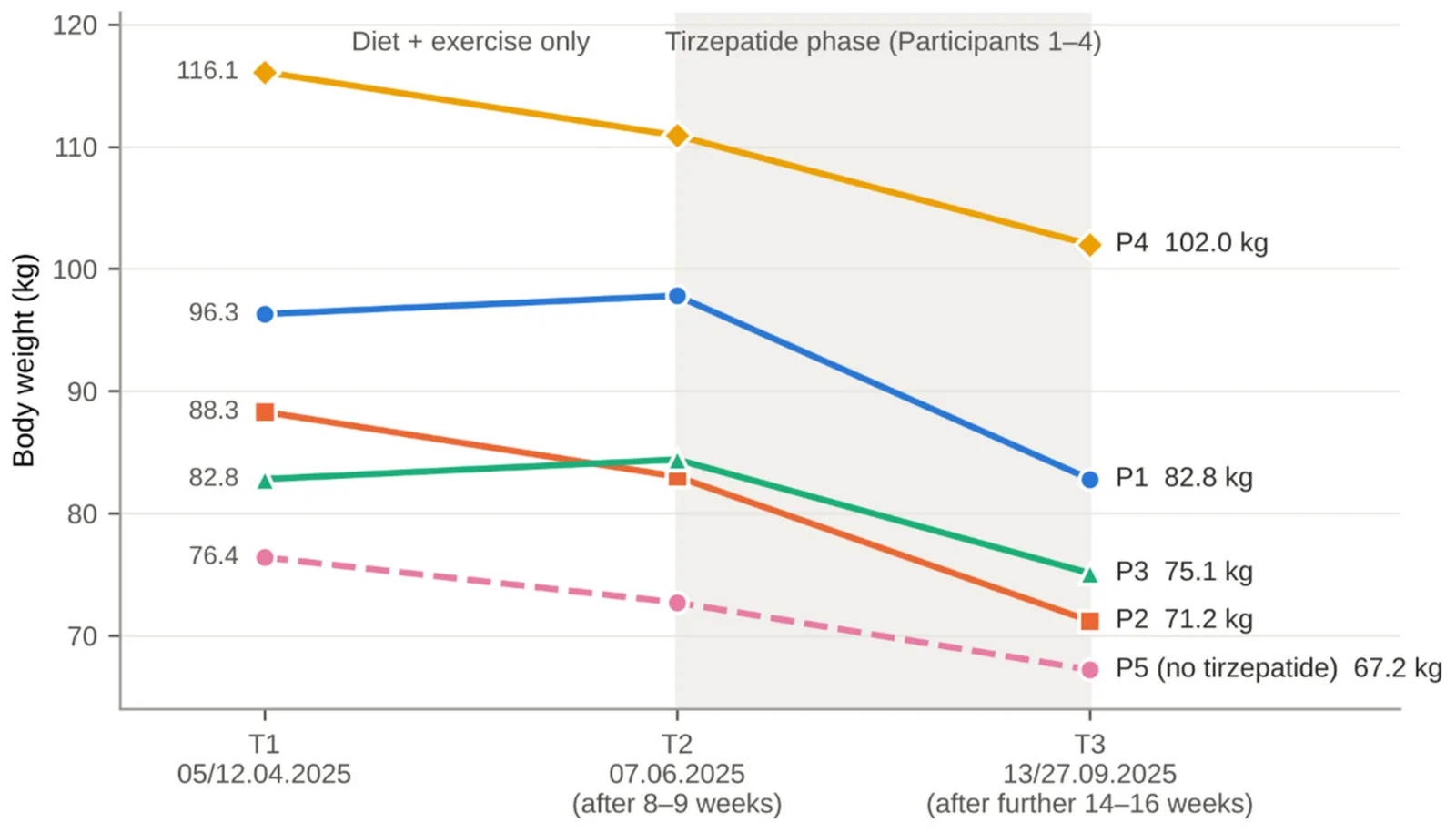
Individual body-weight trajectories at T1 (baseline), T2 (after 8–9 weeks; tirzepatide initiation in Participants 1–4), and T3 (14–16 weeks after T2). Participant 5 did not receive tirzepatide.

**Figure 4:**
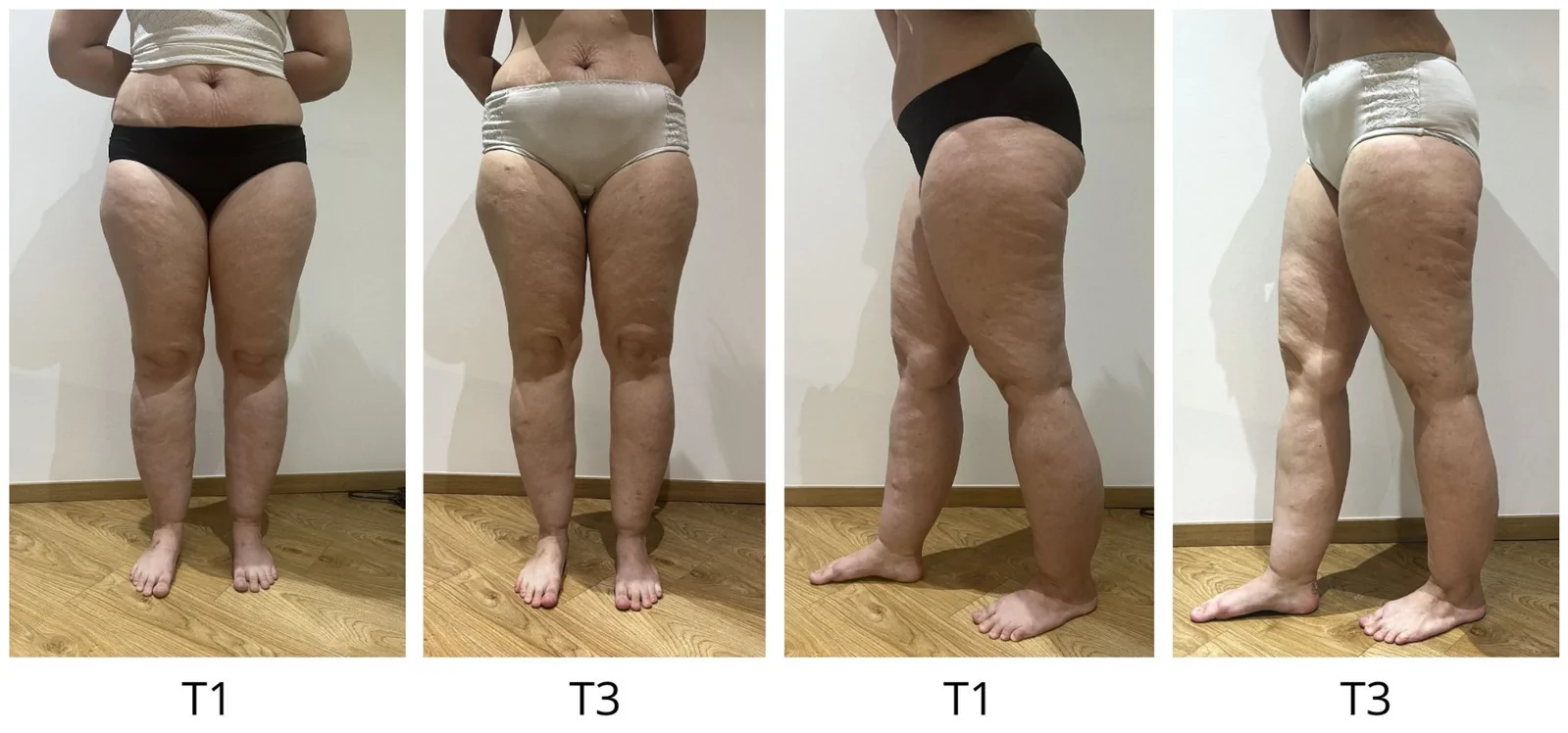
Participant 1 before and after clinical intervention. T1 = week 0; T3 = week 22–25.

**Figure 5:**
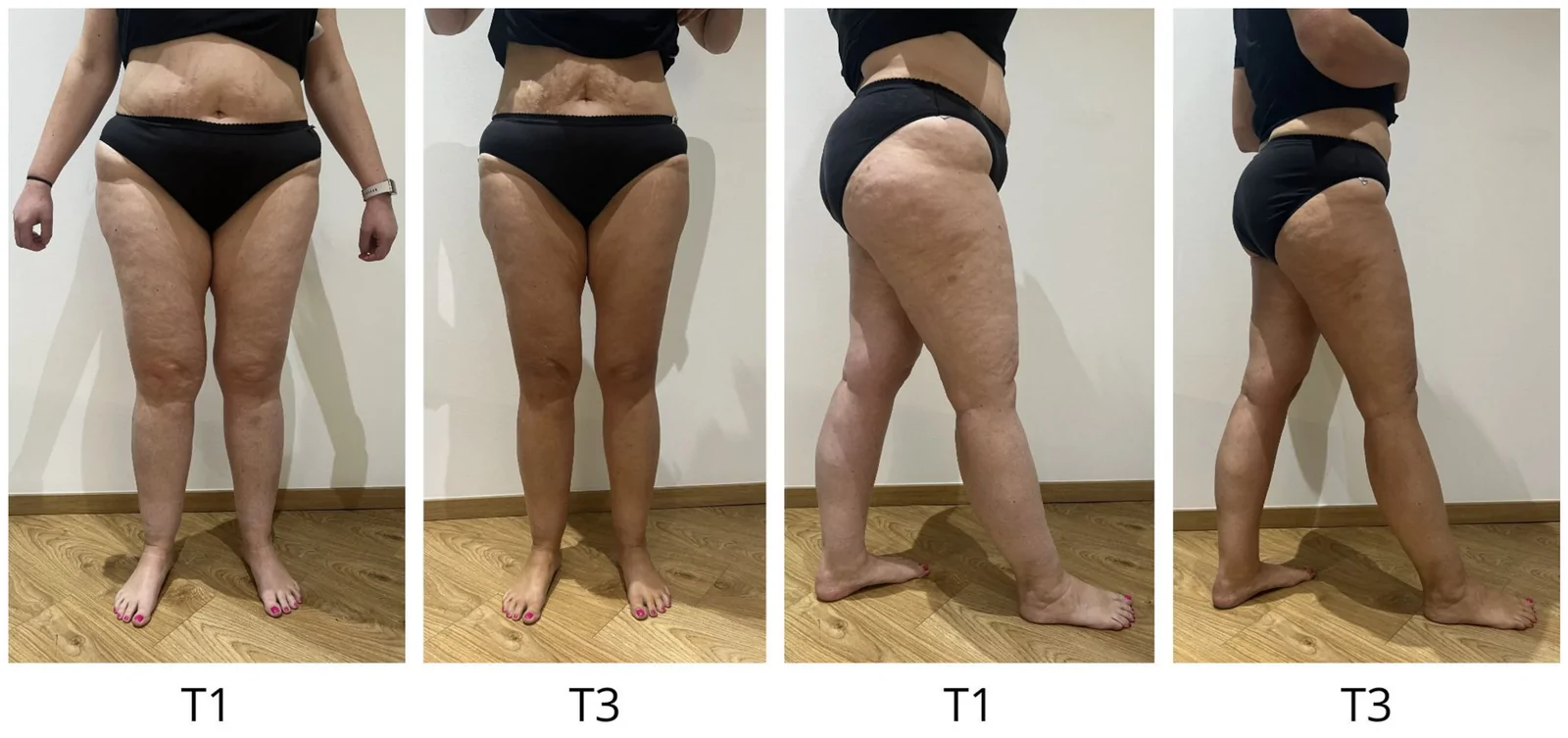
Participant 2 before and after clinical intervention. T1 = week 0; T3 = week 22–25.

**Figure 6:**
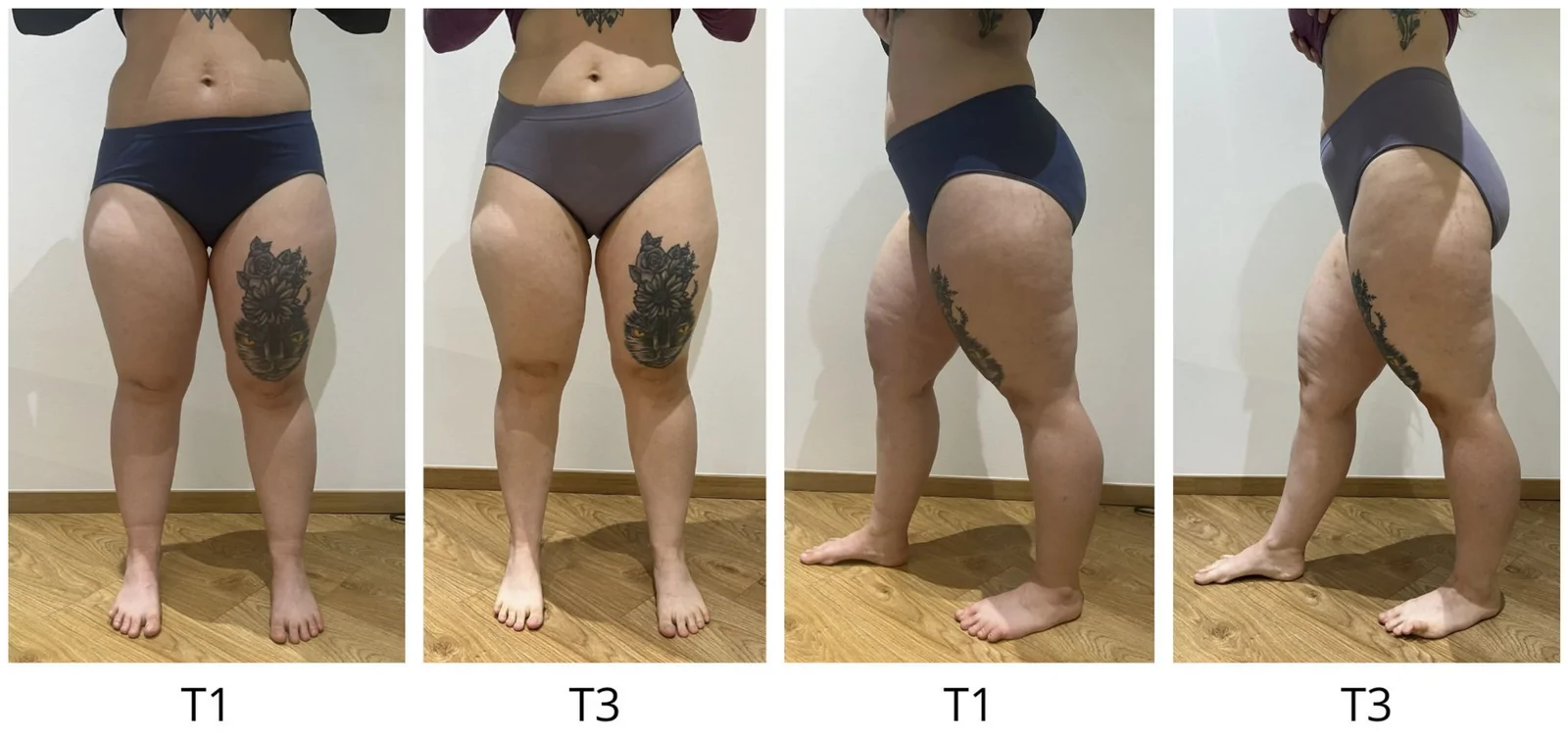
Participant 3 before and after clinical intervention. T1 = week 0; T3 = week 22–25.

**Figure 7:**
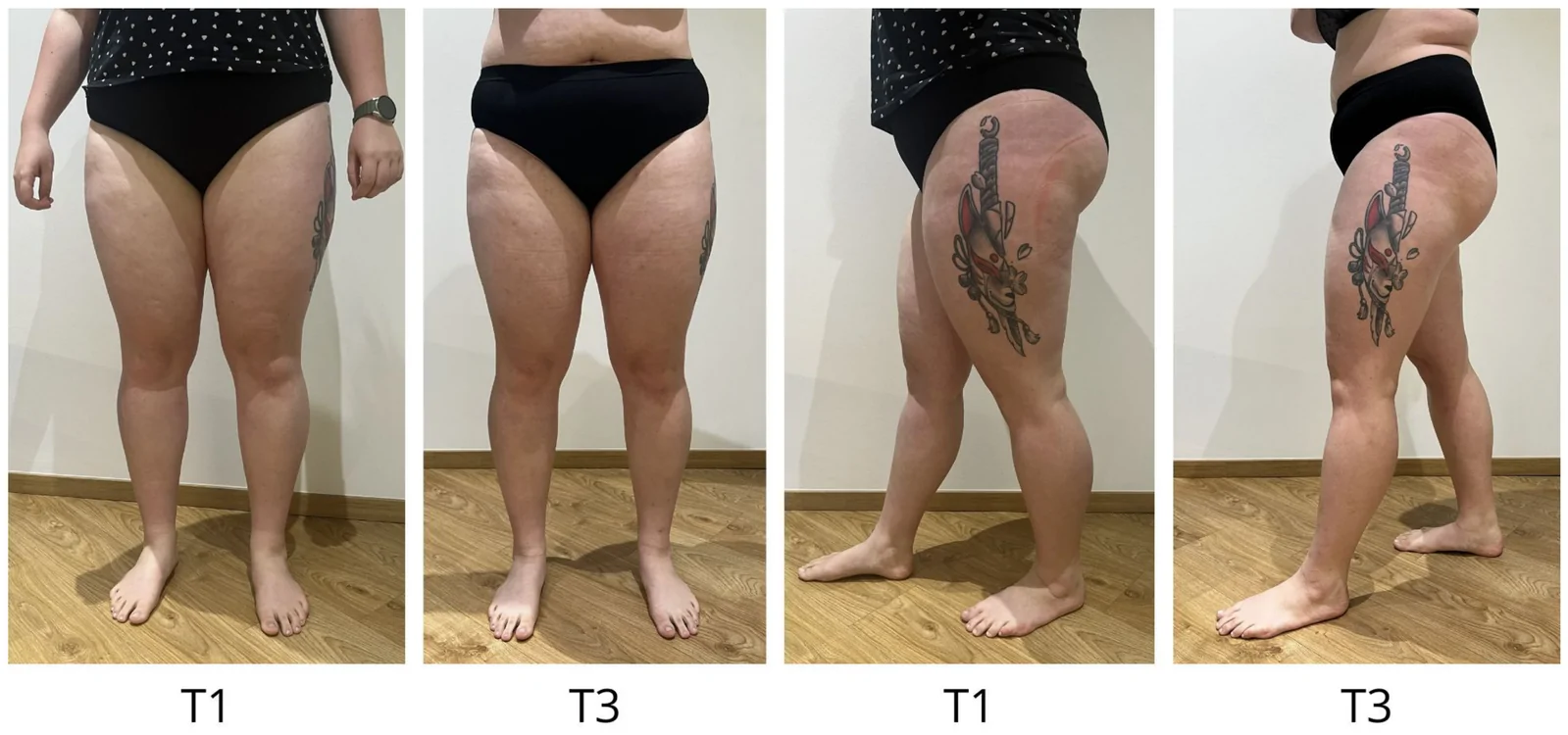
Participant 4 before and after clinical intervention. T1 = week 0; T3 = week 22–25.

**Figure 8:**
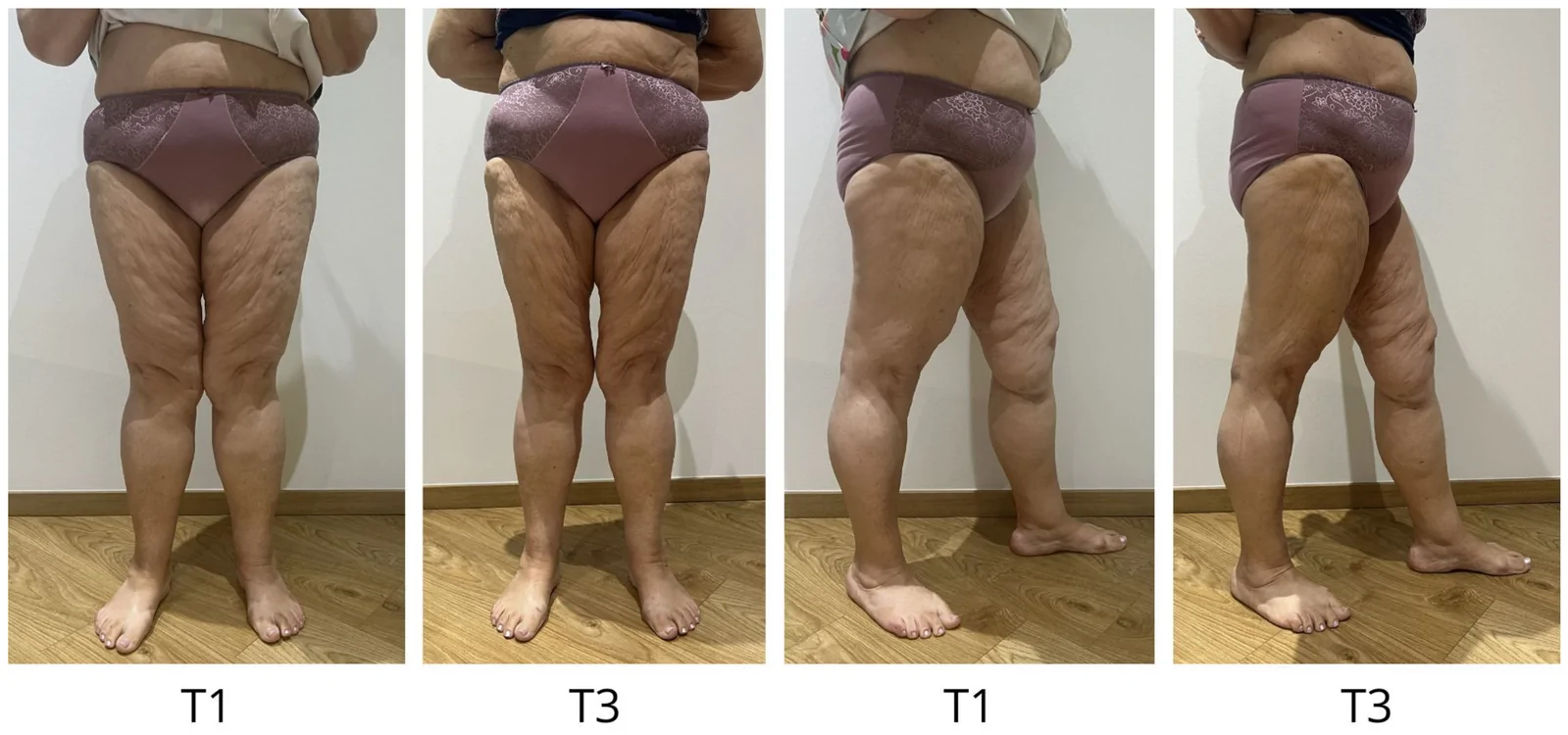
Participant 5 before and after clinical intervention. T1 = week 0; T3 = week 22–25.

**Table 1:** Participant-level timeline (dates as dd.mm.yyyy).

	T1	T2 (Tirzepatide Start)	T3	Verbal Consent (2026 Follow-Up Visit)
Participant 1	05.04.2025	07.06.2025	13.09.2025	26.03.2026
Participant 2	05.04.2025	07.06.2025	27.09.2025	07.04.2026
Participant 3	05.04.2025	07.06.2025	13.09.2025	17.03.2026
Participant 4	12.04.2025	07.06.2025	13.09.2025	29.04.2026
Participant 5	05.04.2025	07.06.2025 (no tirzepatide)	27.09.2025	10.04.2026

T1–T3: routine clinical care (2025); dates from the InBody 570 records. Bioethics Committee approval: 4 February 2026 (Resolution No. 12/2026). Verbal consent to further follow-up was given at each participant’s follow-up visit after approval and recorded in the medical documentation; written consent for publication of data and photographs was signed in October 2026.

**Table 2:** Characteristics of the five individual participants. T1 denotes week 0, before the 8–9-week diet-and-exercise-only phase; T2 denotes week 8–9, immediately before tirzepatide initiation in Participants 1–4; and T3 denotes week 22–25, after 14–16 weeks of tirzepatide exposure.

	8–9-Week Diet/Exercise Phase, Then Tirzepatide (Weeks 8–9 to 22–25)				Diet and Exercise Throughout; no Tirzepatide
Characteristic	Participant 1	Participant 2	Participant 3	Participant 4	Participant 5
Age (years)	30	45	36	26	60
Lipedema type	III and IV	III	II	II	II
Lipedema stage (lower limbs)	2	2	1	2	2
Chronic venous disease	Yes	Yes	No	Yes	No
CEAP classification	C2	C2	C0	C1	C1
Fovea sign (pitting edema)	Negative	Negative	Negative	Negative	Negative
Stemmer’s sign	Negative	Negative	Negative	Negative	Negative
Easy bruising	Yes	Yes	Yes	Yes	Yes
Leg heaviness/fatigue	Yes	No	Yes	Yes	Yes
Pressure pain	Present	Present	Present	Present	Present
Joint pain	No	No	No	No	Yes

Lipedema type: I = buttocks and hips; II = buttocks to knees; III = buttocks to ankles. Lipedema stage: 1 = smooth skin; 2 = uneven/dimpled skin. CEAP classification: C0 = no visible venous disease; C1 = telangiectasias/reticular veins; C2 = varicose veins. Fovea sign: indentation persisting after pressure (indicates lymphedema when positive). Stemmer’s sign: inability to pinch skin fold at base of second toe (indicates lymphedema when positive). BMI, body fat, and skeletal muscle mass have been moved to Table 3.

**Table 3:** Body weight and body composition (InBody 570) at T1, T2, and T3, with changes during the diet-and-exercise phase (T1–T2), the subsequent phase (T2–T3; tirzepatide in Participants 1–4), and overall (T1–T3).

	Participant 1	Participant 2	Participant 3	Participant 4	Participant 5 (No Tirzepatide)
**Body weight (kg)**					
T1	96.3	88.3	82.8	116.1	76.4
T2	97.8	83.0	84.4	110.9	72.7
T3	82.8	71.2	75.1	102.0	67.2
Δ T1–T2	+1.5	−5.3	+1.6	−5.2	−3.7
Δ T2–T3	−15.0	−11.8	−9.3	−8.9	−5.5
Δ T1–T3	−13.5	−17.1	−7.7	−14.1	−9.2
**BMI (kg/m^2^)**					
T1	31.8	32.4	30.4	38.3	32.2
T2	32.3	30.5	31.0	36.6	30.7
T3	27.3	26.2	27.6	33.7	28.3
Δ T1–T2	+0.5	−1.9	+0.6	−1.7	−1.5
Δ T2–T3	−5.0	−4.3	−3.4	−2.9	−2.4
Δ T1–T3	−4.5	−6.2	−2.8	−4.6	−3.9
**Body fat (%)**					
T1	42.5	47.3	41.3	45.9	47.4
T2	42.2	43.8	42.1	45.4	43.3
T3	38.6	38.4	42.4	44.2	40.5
Δ T1–T2	−0.3	−3.5	+0.8	−0.5	−4.1
Δ T2–T3	−3.6	−5.4	+0.3	−1.2	−2.8
Δ T1–T3	−3.9	−8.9	+1.1	−1.7	−6.9
**Fat mass (kg)** *					
T1	40.9	41.8	34.2	53.3	36.2
T2	41.3	36.4	35.5	50.3	31.5
T3	31.9	27.4	31.9	45.0	27.2
Δ T1–T2	+0.4	−5.4	+1.3	−3.0	−4.7
Δ T2–T3	−9.4	−9.0	−3.6	−5.3	−4.3
Δ T1–T3	−9.0	−14.4	−2.3	−8.3	−9.0
**Skeletal muscle mass (kg)**					
T1	30.4	25.5	26.5	35.1	21.3
T2	31.0	25.7	26.7	33.8	21.9
T3	27.8	23.9	23.3	31.5	21.2
Δ T1–T2	+0.6	+0.2	+0.2	−1.3	+0.6
Δ T2–T3	−3.2	−1.8	−3.4	−2.3	−0.7
Δ T1–T3	−2.6	−1.6	−3.2	−3.6	−0.1

Δ = change (later minus earlier value). Measurement dates: T1 05.04.2025 (Participants 1, 2, 3, and 5) and 12.04.2025 (Participant 4); T2 07.06.2025; T3 13.09.2025 (Participants 1, 3, and 4) and 27.09.2025 (Participants 2 and 5). BMI at T2 was calculated from body weight and height. * Fat mass at T1 and T2 calculated as body weight × body fat percentage; at T3 as reported by the analyzer. Participant 5 continued diet and physical activity alone throughout.

**Table 12:** Follow-up observations after T3.

	Date of Contact	Data Source	Observation
Participant 1	14.09.2026	Telephone and e-mail	Body weight 66 kg (reported); tirzepatide discontinued (last dose 2.5 mg on 21 July 2026); continuing diet and physical activity without pharmacotherapy.
Participant 2	07.04.2026	Clinic visit	Tirzepatide 2.5 mg once weekly; following the diet and physical-activity plan.
	06.08.2026	Clinic visit	Cumulative weight loss of 24 kg and increase in skeletal muscle mass of approximately 2 kg reported; tirzepatide being tapered; no adverse effects reported; previously identified prediabetes no longer present; blood count and lipid panel within normal limits; hs-CRP 0.47 mg/L; ACR 2.86 mg/g; eGFR >90 mL/min/1.73 m^2^; mild hepatic steatosis on imaging.
Participant 3	18.12.2025	Clinic visit	Body weight 71.1 kg; tirzepatide 7.5 mg once weekly; improvement in lipid parameters and no adverse effects reported; partial adherence to diet and physical activity.
	14.09.2026	Telephone and e-mail	Body weight 67 kg; tirzepatide reduced to 5 mg once weekly; following the diet and physical-activity plan.
Participant 4	29.04.2026	Clinic visit	Tirzepatide 7.5 mg once weekly, well tolerated; body weight 95 kg; improvement in dyslipidemia reported; levothyroxine 50 µg daily, vitamin D3, and omega-3-6-9 continued.
	14.09.2026	Telephone and e-mail	Tirzepatide 7.5 mg once weekly; following the diet and physical-activity plan.
Participant 5	14.09.2026	Telephone and e-mail	Body weight 59.4 kg; continuing diet and physical activity; no pharmacotherapy at any time.

## Data Availability

The data presented in this study are available on reasonable request from the corresponding author. The data are not publicly available because they contain individual health information that could compromise the privacy of the small number of participants.

## Supplementary Materials

The following supporting information can be downloaded at: https://www.mdpi.com/article/10.3390/jcm15218643/s1, Table S1: Central Circumferences and Anthropometric Ratios.

## Clinical Trial Registration

This project was not conducted as a randomized clinical trial or as a clinical trial of an investigational medicinal product. Tirzepatide was initiated after the 8–9-week non-pharmacological phase and was prescribed within its approved obesity indication rather than as an investigational therapy for lipedema. The study is observational (ambispective): tirzepatide and all other treatments were prescribed in routine clinical care, and participants were not assigned to any intervention for research purposes. Registration in a clinical trial registry was therefore not required.
