# Supplementary material for: A Multimodal Intervention Including Tirzepatide in Women with Lipedema and Excess Body Weight: A Pilot Case Series
Source: J Clin Med. 2026 Nov 6;15(21):8643. doi: 10.3390/jcm15218643 (PMC13763609; doi:10.3390/jcm15218643)
Supplement: Supplementary file 1 [file jcm-15-08643-s001.zip › jcm-4596232-supplementary.pdf]

Supplementary material

Table S1. Central Circumferences and Anthropometric Ratios. T1 denotes week 0, before the 8–9-week diet-and-exercise-only phase; T2 denotes week 8–9, immediately before tirzepatide initiation in Participants 1–4; T3 denotes week 22–25, after 14–16 weeks of tirzepatide exposure in Participants 1–4 (Participant 5 received the non-pharmacological intervention alone throughout the observation period).

Characteristic	**Participant 1**	**Participant 2**	**Participant 3**	**Participant 4**	**Participant 5**
Height (cm)	174	165	165	174	154
Waist circumference (cm)					
T1/T2/T3	86.0<br>85.0<br>85.0	96.5<br>92.0<br>81.5	82.0<br>80.0<br>75.0	106.0<br>101.0<br>98.0	94.0<br>94.0<br>88.5
Hip circumference (cm)					
T1/T2/T3	122.0<br>121.5<br>117.0	120.0<br>117.0<br>108.0	122.0<br>119.0<br>114.5	127.0<br>128.0<br>121.5	105.5<br>105.0<br>102.5
WHR					
T1/ T2/T3	0.70<br>0.70<br>0.73	0.80<br>0.79<br>0.75	0.67<br>0.67<br>0.66	0.83<br>0.79<br>0.81	0.89<br>0.90<br>0.86
WHtR					
T1/T2/T3	0.49<br>0.49<br>0.49	0.58<br>0.56<br>0.49	0.50<br>0.48<br>0.45	0.61<br>0.58<br>0.56	0.61<br>0.61<br>0.57

WHR = Waist-to-Hip Ratio (>0.85 in women indicates abdominal obesity); WHtR = Waist-to-Height Ratio (>0.5 indicates increased cardiometabolic risk).
